# Managing Sex as a Biological Variable in Physiological Research: Best Practices

**DOI:** 10.1093/function/zqae034

**Published:** 2024-08-05

**Authors:** Candee T Barris, Emily Burns-Ray, Jennifer C Sullivan

**Affiliations:** Vascular Biology Center, Department of Physiology, Medical College of Georgia, Augusta University, Augusta, GA 30912, USA; Vascular Biology Center, Department of Physiology, Medical College of Georgia, Augusta University, Augusta, GA 30912, USA; Vascular Biology Center, Department of Physiology, Medical College of Georgia, Augusta University, Augusta, GA 30912, USA

Eight years after the historic NIH policy (NOT-OD-15-102) mandating the consideration of sex as a biological variable (SABV) in preclinical biomedical research, scientists are still struggling with the practical and appropriate incorporation of SABV. The importance of SABV was further highlighted this March when the White House issued an executive order prioritizing the advancement of women’s health research to address the unmet needs of the diseases and conditions that disproportionately affect women across the lifespan.^1^ When SABV is factored into the design, analysis, and reporting of biomedical research, it is not simply addressing a women’s health issue, but ultimately addressing the issue of ensuring rigorous and reproducible data-driven science aimed at providing better health for all. SABV inclusion is essential to narrow and/or close the gaps of knowledge, identify under-addressed questions in the field, and revisit our own scientific assumptions and biases based upon previous, single-sex-driven research. This call to action was recently followed by the 18th annual meeting of the Organization for the Study of Sex Differences (OSSD), where investigators gathered to present research focused on investigating sexual dimorphisms in disease. This gathering was a fortuitous opportunity to discover challenges faced and techniques used to incorporate SABV.

Firstly, it is crucial that we define sex and gender. These terms are often used interchangeably, which confounds data and ultimately leads to inaccurate reporting. *Sex* refers to the biological classification based on sex chromosome complement, whereas *gender* falls within a spectrum of socially constructed roles and identities. Both sex and gender must be acknowledged in research. Scientists have become increasingly aware of the role of sex and its effects on research, but one must not undervalue the role of gender, as it interacts with and influences biology, health, and disease. Although gender is an integral aspect to SABV and a critical consideration in research, it is a term that is exclusively applicable to human studies, as it a unique trait of self-concept and social construct not applicable to non-human laboratory animals. Proper use of the terms will enhance accuracy of meta-analysis and prevent obfuscated results.

Biological sex, encompassing both genetic and hormonal differences between males and females,^2^ can profoundly influence the onset, progression, and treatment outcomes of disease. Despite this, a tendency to over-generalize data derived from experimental models utilizing males persists, leading to inconsistent assumptions regarding women’s health and detrimental consequences for women regarding treatment of disease in clinical practice. Consideration of SABV allows for scientists to uncover divergent sex-specific risk factors, pathophysiological mechanisms, disease progression, and treatment responses. Deliberate and transparent reporting of sex-specific experimental details and data improves health outcomes for all individuals by enabling the development of more personalized and effective interventions that account for the inherent biological differences of women versus men.

## SABV Best Practices

Ironically, when females are included in experimental design, beyond the scope of pregnancy studies, researchers often utilize virgin animals and avoid accounting for estrous cycling variability. This is an acceptable starting point, but it should be recognized that limitations persist—for example, many of the women who present with cardiovascular disease and receive treatment intervention are multiparous and postmenopausal.^3^ A 2021 examination of published sex differences across multiple disciplines reports missing statistical evidence and misinterpretation of results, suggesting that although researchers are now testing for sex differences, they may not be adequately equipped or trained to do so correctly.^4^ This was most robustly seen by the lack of statistical analyses to directly compare responses in males and females or, conversely, where potential differences were masked by pooling data without initially testing to see whether sex differences existed. These are just a few of the common missteps made by investigators. To begin to properly incorporate sex as a factor in preclinical biomedical research, start with the following best practices for robust implementation of SABV to strengthen physiological research:

1. *Determine whether the area of research has  implications  for both males and females*. Human conditions do not always warrant the study of both sexes. Some diseases are markedly different between men and women, affecting one sex uniquely or disproportionately. If the physiology or pathophysiology being investigated does have sex differences, best practice would then dictate that the investigator should account for the influence of sex in the experimental design, consider whether sex should be a factor in the hypothesis, and include both males and females in the experimental design. If the investigatory question is more appropriate for one sex, provide adequate justification for studying a single sex.
2. *Power the study appropriately and analyze the data to allow for sex-specific conclusions to be drawn*. In order to be able to determine the presence of sex differences, the experimental design must be adequately powered in order to detect them. Moreover, data should be desegregated and analyzed to directly compare findings in males and females. If no difference is observed, this too should be reported and only then is it appropriate to consider pooling the data.
3. *Report data  separated by sex*. It is not enough to simply include both sexes in your experimental design. Communicating your results with the data graphed to allow for sex-specific reporting is critical to increase transparency and increase the likelihood that the population most likely to benefit or be impacted by the findings is clear. Researchers should clearly state the limitations of the study and clarify whether the studies do not test for sex differences. It is also important for investigators to report null findings. Reporting the absence of a difference is imperative to a better understanding of disease, prevention of wasted funds, resources, and most importantly, to improve human health by better reported science.

## SABV for the Reviewer

A comprehensive understanding of these best practices will also greatly benefit peer review. Reviewers need to be cognizant of how to best address SABV, such that requests for revisions are realistic and reasonable. Since the release of the NIH mandate to consider SABV, reviewers have been tasked with assessing its inclusion in grant proposals and journal submissions, thereby holding the primary responsibility to evaluate the investigator’s intent to consider SABV. Despite NIH providing Reviewer Guidance of SABV, some reviewers still ignore the issue, while others misunderstand its practical application. Reviewers who do not understand the appropriate and logical consideration of SABV have therefore inaccurately assumed that SABV means both sexes must always be included, which perpetuates this misunderstanding to investigators. Reviewers have unreasonably asked for other sex to be included in studies where it makes no scientific sense, provoking a myriad of frustrations from researchers. Peer-review scores should recognize investigators who rigorously consider SABV, but this cannot be accurately done without adequate understanding of SABV guidance. If reviewers have a sound grasp of the best practices listed above, it will help to improve the peer-review process.

In summary, future studies are generally based on past results, but the lack of the inclusion of females in research has resulted in women being at a significant therapeutic disadvantage. This needs to change. Men and women have inherent biological differences, necessitating a shift from “Bikini Medicine,” the misconception that women’s health research is limited to areas covered by a bikini, an approach that is no longer appropriate and is ultimately detrimental. Adhering to the NIH’s SABV guidance is imperative to ensure better human health for all, not just half the human population. While sex is not a causal mechanism nor is it relevant for every research question, careful consideration should be taken when investigators approach and design experiments. A thoughtful lens should be utilized when making assumptions about sex differences in (patho)physiology and these differences must be described with appropriate language.
